# Evolution in the Diagnosis and Treatment of Myocarditis in Recent Years: State of the Art

**DOI:** 10.3390/jcm14217661

**Published:** 2025-10-28

**Authors:** Jeness Campodonico, Chiara Lauri, Beatrice Pezzuto, Piergiuseppe Agostoni, Carlo Vignati

**Affiliations:** 1 Department of Critical Cardiology and Rehabilitation, Centro Cardiologico Monzino, IRCCS, 20138 Milan, Italychiaralauri.mail@gmail.com (C.L.); beatrice.pezzuto@cardiologicomonzino.it (B.P.); piergiuseppe.agostoni@cardiologicomonzino.it (P.A.); 2 Department of Medicine and Surgery, University of Insubria, 21100 Varese, Italy; 3 Department of Clinical Sciences and Community Health, Cardiovascular Section, University of Milano, 20122 Milan, Italy

**Keywords:** myocarditis, cardiac magnetic resonance imaging, endomyocardial biopsy, therapy, cardiomyopathy

## Abstract

Acute myocarditis (AM) is an inflammatory cardiac condition resulting from infections, toxic exposures, or immune-mediated mechanisms, with clinical presentations ranging from mild symptoms to heart failure (HF) or cardiogenic shock. Although viral infections remain the predominant cause, both the absolute prevalence and the relative distribution of different etiologies may change over time and across regions depending on endemic diseases. Immune checkpoint inhibitor (ICI)-associated myocarditis has emerged as a newly recognized entity, with diagnostic rates increasing in parallel with growing awareness and the expanding population of cancer patients eligible for ICI therapy. Additionally, genetic predisposition—particularly mutations linked to arrhythmogenic cardiomyopathy—is also being increasingly acknowledged as a susceptibility factor. Recent advances have markedly improved the diagnostic approach to AM. The availability of high-sensitivity cardiac troponins and the widespread use of cardiac magnetic resonance imaging (CMRI) have enhanced early detection and tissue characterization. CMRI, especially following the updated Lake Louise Criteria (2018), which incorporate T1 and T2 mapping, enables accurate assessment of myocardial inflammation and fibrosis. Endomyocardial biopsy (EMB) remains essential in complicated cases, particularly to identify histologic subtypes that may benefit from immunosuppressive therapy. Early EMB (within 48 h) has been associated with better outcomes in fulminant presentations. The use of immunohistochemistry with leukocyte-specific markers has further increased the sensitivity of EMB. Therapeutic strategies now integrate etiology-specific approaches. Immunosuppressive therapy is indicated for distinct histological forms such as eosinophilic (EM) and giant cell myocarditis (GCM) or cases associated with systemic autoimmune disease. Conversely, in most patients with acute myocarditis complicated by acute HF or cardiogenic shock, no specific treatment is currently recommended beyond evidence-based management of acute HF and general supportive therapy.

## 1. Introduction

Myocarditis is an inflammatory disease of the myocardium responsible for clinical presentations of varying severity, often found in post-mortem reports of individuals who died from sudden cardiac death (SCD), predominantly men under the age of 35 [1]. In most cases, it follows an uncomplicated course; however, it can lead to acute heart failure (HF), cardiogenic shock, and fatal arrhythmias. Long-term complications primarily include progressive left ventricular (LV) dysfunction and recurrent arrhythmias. The etiology is diverse, encompassing infectious, autoimmune, pharmacological causes, and anticancer treatments, especially following the introduction of immune checkpoint inhibitors (ICI). The diagnostic and therapeutic approach varies according to the clinical presentation. While endomyocardial biopsy (EMB) was once necessary for the diagnosis of myocarditis, it is now reserved for high-risk cases requiring early histological type definition and immunosuppressive therapy initiation. Multimodal imaging has become a cornerstone in myocarditis diagnosis, with cardiac magnetic resonance imaging (CMRI) playing a crucial role. Furthermore, there is emerging evidence of a correlation between genetic mutations, responsible for cardiomyopathies, and recurrent forms of acute myocarditis (AM), which are associated with increased late gadolinium enhancement (LGE) on CMRI and a higher arrhythmic risk. Pharmacological treatment is mainly supportive, though corticosteroid therapy and immunosuppressive treatment are indicated for specific histological subtypes. This review aims to provide an updated overview of the current state of the art in the diagnosis and treatment of AM, considering new recommendations, technological advances, and changes in both epidemiology and etiology, also in light of the most recent European guidelines on myocarditis and pericarditis [2]. AM is a condition that primarily affects young individuals and can lead to fatal outcomes in both the short and long term. Therefore, addressing the existing gaps in its management is of critical importance.

## 2. Epidemiology

Myocarditis predominantly affects young individuals, with a median age ranging between 20 and 40 years [3,4,5,6]. In contrast, drug-induced forms (e.g., ICIs and clozapine) are more frequently observed in the elderly [7,8]. AM occurs more often in males, accounting for approximately 75–84% of cases. Conversely, in fulminant myocarditis (FM), the male-to-female ratio appears to be more balanced [3,9,10].

The epidemiology of AM has been difficult to determine over the years due to the percentage of cases that are asymptomatic or lead to death before diagnosis, along with changes in diagnostic algorithms. According to the 2013 European guidelines, EMB was recommended for a definitive diagnosis of myocarditis [11], though it was performed only in selected cases. With advances in imaging techniques, CMRI has gained increasing importance, enabling non-invasive diagnosis [2]. The percentage of AM cases has increased from 5% to 13% with the use of CMRI and high-sensitivity troponin (hs-Tn) in patients with angina-like symptoms [12]. Data from the global burden of cardiovascular disease between 1990 and 2019 report a prevalence of 4.2–8.7 per 100,000 people in the 35–39 age group [13]. Autopsy studies on sudden unexplained death in the young report histologically confirmed myocarditis prevalence ranging from 2% to 42% of cases [1]. EMB-proven myocarditis has been reported in 9–16% of adults with non-ischemic dilated cardiomyopathy and up to 46% of pediatric cases with dilated cardiomyopathy [14].

## 3. Etiology

Myocarditis can have either infectious or non-infectious etiology. In genetically predisposed individuals, exposure to a triggering factor can lead to myocardial damage via autoimmune/inflammatory mechanisms. The most common etiology appears to be infectious, with enteroviruses, adenoviruses, parvovirus B19 (B19V), and some herpesviruses (Epstein–Barr virus (EBV) and Herpes Virus 6 (HHV6)) being the most frequently encountered, along with influenza and coronaviruses [15,16,17,18]. The persistence of viral genome in the myocardium is associated with progressive worsening of LV function [19]. AM due to B19V often presents as an infarct-like syndrome and has a generally favorable long-term prognosis, although fatal cases have been reported. Myocarditis due to HHV-6, particularly co-infection with HHV-6 and B19V, often leads to acute HF symptoms and frequently progresses to chronic HF [20].

**COVID-19-associated myocarditis.** The COVID-19 pandemic has led to numerous myocarditis cases, although it remains unclear whether SARS-CoV-2 causes direct myocardial injury. Several mechanisms have been proposed to explain myocardial injury temporally linked to SARS-CoV-2 infection, including thromboembolic events, activation of inflammatory pathways such as cytokine storms, and autoimmune reactions [21,22,23]. Autopsy studies have frequently reported low viral loads within the myocardium, raising questions about the direct cytotoxic effects of SARS-CoV-2 [24,25,26,27,28]. Clinically, COVID-19-associated myocarditis resembles other viral forms of myocarditis [29], and its diagnosis should follow standard diagnostic algorithms [2]. In addition to confirming AM, CMRI can detect concurrent ischemic damage, microinfarctions, and right ventricular (RV) involvement [30,31,32]. COVID-19-associated myocarditis may also manifest as part of a multisystem inflammatory syndrome, typically occurring 4–5 weeks after initial infection [2]. This form often shows diffuse myocardial inflammation on CMRI and is more prevalent in pediatric populations than in adults [33,34]. Management primarily involves antiviral therapy and supportive care, with vasopressors, inotropes, or corticosteroids as needed [2]. Although immunosuppressive therapies have shown benefit in critically ill COVID-19 patients, their role in myocarditis specifically remains unproven, and corticosteroid use in severe cases is still debated [2,35]. Cases of CMR-confirmed myocarditis following COVID-19 vaccination have been reported, with incidence varying by vaccine type, dose, age, and sex (0.34–3.72 per 100,000), more frequently in young males after the second dose [36,37,38,39,40]. Presentations are generally mild, outcomes are favorable [41,42,43], and diagnosis and treatment should follow standard myocarditis protocols, as no specific therapies have been established [2].

Among bacterial infections, Lyme disease caused by *Borrelia burgdorferi* is the most notable, while Chagas disease, caused by the *Trypanosoma cruzi* parasite, is endemic in certain regions of the world [2].

Non-infectious causes are also numerous, including autoimmune diseases, immune-mediated forms (characterized by histopathological findings such as lymphocytic myocarditis (LM), giant cell myocarditis (GCM), eosinophilic myocarditis (EM), and cardiac sarcoidosis (CS)), inflammatory bowel diseases, chest-radiation, chronic inflammatory cardiomyopathies (infl-CMP) due to genetic conditions [44], and drug or toxin reactions [20]. Among the latter, ICI-associated myocarditis has emerged in recent years. These monoclonal antibodies target specific molecules that inhibit immune response (e.g., CTLA-4, PD-1, PDL-1, and LAG-3), enhancing T-cell responses against cancer. It affects approximately 1% of treated patients with early onset after treatment initiation (median 30 days) and a mortality rate of up to 50%. The risk increases with combination therapy using two types of ICIs. With the growing number of patients eligible for ICI therapy, the absolute number of ICI-induced myocarditis cases is rising, though it remains a rare complication [45].

## 4. Clinical Scenarios

The recent European Society of Cardiology (ESC) guidelines on the management of myocarditis and pericarditis [2] have adopted a clinical approach based on temporal criteria and clinical presentation, which are useful for identifying high-risk cases.

From a temporal perspective, myocarditis is characterized by different phases: acute, subacute, and chronic (Table 1) [2].

The course of the disease is variable among patients, and not all phases are necessarily experienced. Some patients may recover completely without sequelae, while others may develop complications, which in some cases can lead to death.

**Acute myocarditis.** The onset of the disease is defined as acute if symptoms occurred within the last 4 weeks. This scenario also includes FM [3]. AM can present with symptoms and signs of varying severity, ranging from oligosymptomatic forms to life-threatening conditions [2]. Chest pain is the most frequent symptom in adolescents and adults, occurring in more than 80% of cases. Dyspnea occurs in 20–50% of cases, whereas other symptoms, including fatigue, palpitations, and syncope, are less frequent, reported in approximately 5% of patients [3,4,46,47]. In the majority of cases (80%), symptoms are preceded by prodromal manifestations such as fever (60%), gastrointestinal complaints (30%), or respiratory symptoms (25%) [3,4,48]. FM (3–9% of cases) is characterized by rapid hemodynamic deterioration leading to cardiogenic shock, requiring inotropic and/or mechanical circulatory support [3]. Isolated right ventricular dysfunction is rare, while biventricular dysfunction is more common. These patients may present with sustained arrhythmias (46.9%) and, in some cases, may present with SCD (25.8%) [3,48,49].

**Subacute myocarditis.** The subacute stage refers to cases in which symptoms persist for more than 1 month but no longer than 3 months [44].

**Chronic myocarditis.** Myocarditis is considered chronic when symptoms extend beyond 3 months [44]. Chronic myocarditis may represent an intermediate stage progressing to inflammatory cardiomyopathy, characterized by ventricular dysfunction and cardiac remodeling [11,44]. Milder forms can present as non-dilated hypokinetic cardiomyopathy [44]. In these patients, arrhythmic risk should be considered, given the presence of fibrosis [9,44].

Myocarditis is characterized by heterogeneity of clinical manifestations with different phenotypes. A clinical classification distinguishing high-risk from non-high-risk myocarditis has been shown to reliably predict outcomes in large patient cohorts [2,3,50] (Table 2).

The recent ESC guidelines have proposed distinct diagnostic algorithms based on the presence of red flags for the clinical diagnosis of myocarditis (Table 3), and the clinical presentation (acute chest pain, acute heart failure, and new, recurrent, or unexplained arrhythmias) [2].

**Pseudo-infarction phenotype.** Most cases have a pseudo-infarction presentation, with acute chest pain, electrocardiographic abnormalities (especially ST-segment elevation), and increased necrosis biomarkers, particularly troponin [51], which today is able to identify more low-risk myocarditis cases, thanks to high-sensitivity troponin assays.

**Chest pain phenotype**. Patients presenting with chest pain, preserved LV systolic function, no segmental wall motion abnormalities, absence of VA, and short-term normalization of the electrocardiogram (ECG) can be classified as low-risk, with an excellent long-term prognosis [2,3,50,52,53,54].

**Acute heart failure phenotype.** Patients presenting with acute HF, particularly when associated with left ventricular dysfunction (LVEF ≤ 40%), should be considered high-risk. Prognosis depends on the short-term response to therapy [3,50]. Clinical presentation ranges from unexplained LV dysfunction, with or without ventricular dilation, to new-onset or worsening HF, usually occurring between 2 weeks and 3 months, potentially progressing to refractory HF and cardiogenic shock [20]

**Arrhythmia and atrioventricular block phenotype.** Patients with AM presenting with new-onset, recurrent, or unexplained arrhythmias may exhibit a broad spectrum of symptoms, ranging from palpitations to syncope, or potentially fatal arrhythmias that can result in aborted SCD [11,55]. Patients presenting with advanced atrioventricular block (AVB) or sustained VA should be considered high-risk, regardless of LV systolic function. Additionally, patients with sustained VA in the acute phase have a high risk of recurrence [56]. GCM and CS are more often associated with sustained VA [57,58,59], while advanced AVB is more characteristic of non-viral myocarditis [60]. VA can have various causes, including the presence of inflammatory infiltrates and the release of cytokines, and is usually polymorphic during the acute phase [47]. Ventricular arrhythmias can also be observed in the long-term phase, often related to myocardial fibrosis, and typically present as monomorphic and regular [61]. SCD attributed to myocarditis among patients undergoing autopsy varies from 1.1% to 12%, and males show a higher incidence rate than females [62,63]. Most individuals are asymptomatic before SCD. The presence of LGE in the mid-wall layer of the anteroseptal myocardial segments is associated with a higher risk of mortality and VA [64,65,66]. Septal involvement may confer a worse prognosis due to the presence of a specific genetic background, such as pathogenic variants in desmosomal genes, including desmoplakin (DSP), desmocollin, plakoglobin, and placophillin [67].

**Genetic background.** Over the years, a complex interplay between genetic background, inflammatory response, and autoimmunity has emerged, which appears to be responsible for the heterogeneity of clinical presentation and disease progression in myocarditis. It has been proposed that, in genetically predisposed individuals, a “second-hit” mechanism—such as transient viral infections or mechanical stress—may precipitate recurrent episodes of myocardial inflammation. In pediatric or young adult patients, the first clinical manifestation of the cardiomyopathy often consists of chest pain, associated with ECG changes, elevated cardiac enzymes, and inflammatory markers, mimicking AM [68,69]. A recent meta-analysis by Monda et al. found that causative genetic variants are common in patients with AM, particularly in complicated cases (22% in adults) and in those with early-onset disease (childhood or adolescence) (44%), with sarcomeric gene variants being the most prevalent [70].

Among the pathogenic variants most frequently associated with AM and infl-CMP are those involving DSP and titin (TTN). DSP, a major structural component of the desmosome, mediates the mechanical coupling between desmosomes and intermediate filaments, ensuring cytoskeletal integrity and force transmission. Mechanical stress has been hypothesized to exert a detrimental effect in patients harboring mutations in desmosomal genes (predominantly DSP and DSG2), thereby potentially contributing to inflammatory flares and disease progression. A paradigmatic example is the so-called “hot phase” of arrhythmogenic cardiomyopathy. Patients with DSP-related cardiomyopathy typically exhibit extensive areas of myocardial fibrosis on CMRI, recurrent myocarditis episodes, and, in advanced stages, a high incidence of VA [71]. The initial clinical manifestation may consist of AM, often occurring with preserved LV ejection fraction [67]. Conversely, TTN mutation carriers more frequently present with LV systolic dysfunction, reflecting the pivotal role of TTN in sarcomeric architecture and contractile function [72].

Genetic factors may contribute both to susceptibility and to the modulation of the inflammatory response and myocardial injury, thereby impacting ventricular remodeling and the subsequent development of cardiomyopathy. Additional studies are needed to elucidate this connection. An ongoing clinical trial (ClinicalTrials.gov Identifier: NCT06158698) is currently investigating the impact of a positive genotype in patients with infl-CMP and the effect of colchicine therapy in both genotype-negative and genotype-positive groups. The aim of the study is to explore the interaction between myocardial inflammation and pathogenic genotypes, as well as the potential role of colchicine in reducing inflammation, improving LV systolic function, and decreasing arrhythmic burden, while assessing possible differences in treatment response between genotype-negative and genotype-positive patients.

The diagnostic suspicion of an underlying genetic substrate should arise in cases of recurrent myocarditis, family history of cardiomyopathies, myocarditis, or SCD, the presence of sustained arrhythmias, and patterns of LGE on CMRI (ring-like or septal) [67]. In these cases, genetic testing should be performed (Class II, Level B) [2] (Table 4).

Identification of a pathogenic or likely pathogenic variant in a patient with myocarditis has important implications not only for the patient’s treatment strategy but also for family management, enabling at-risk relatives to be identified through cascade genetic testing. The availability of Next-Generation Sequencing (NGS) has enabled the identification of numerous genetic variants that may be associated with myocarditis, which are still under investigation [47].

**The multi-aspect phenotype.** The so-called complicated forms of myocarditis are characterized by left ventricular ejection fraction (LVEF) < 50% on the first echocardiogram, ventricular arrhythmias (VA), acute HE, and cardiogenic shock. The presence of red flags helps identify high-risk patients, who exhibit a rapidly progressive clinical course and worse prognosis, for whom rapid diagnosis is critical [2] (Table 2).

## 5. Diagnosis

The recent ESC guidelines [2] have revised the diagnostic criteria for myocarditis. According to the 2013 ESC consensus paper [11], EMB was considered the gold standard for diagnosis. However, given the invasiveness of the procedure and the limited number of clinical scenarios in which it is indicated, diagnostic criteria were developed to define the cases where EMB is recommended. These criteria required at least one diagnostic criterion (such as ECG abnormalities/arrhythmias, elevated troponin levels, structural or functional myocardial abnormalities on cardiac imaging and tissue characterization abnormalities on CMRI) in symptomatic patients or at least two of the aforementioned criteria in asymptomatic individuals, in the absence of angiographically detectable coronary artery disease (coronary stenosis ≥ 50%) and known pre-existing cardiovascular disease or extra-cardiac causes that could explain the syndrome [11] (Table 5).

The criteria proposed in the new 2025 ESC guidelines are driven by clinical presentation, with additional supportive findings, and positive CMR or EMB for myocarditis. Advances in multimodal imaging techniques, particularly CMRI, have enabled the non-invasive diagnosis of myocarditis. Myocarditis can be diagnosed clinically as possible or confirmed with appropriate clinical presentation and EMB or CMR, or with an additional criterion (ECG, arrhythmias, echocardiographic findings, biomarkers) in cases where CMRI or EMB are uncertain or unavailable [2]. The diagnosis is excluded if only the clinical presentation is present (Table 6 and Table 7).

**Circulating biomarkers.** Recommended laboratory tests for myocarditis typically include complete blood count, markers of myocardial injury (hs-Tn and CK-MB), and nonspecific inflammatory markers such as C-reactive protein (CRP) and erythrocyte sedimentation rate (ESR). While these tests are widely available and easy to perform, CRP and ESR are nonspecific and may be elevated in any inflammatory condition [73]. Traditional myocardial injury markers often rise in infectious myocarditis but are not exclusive to this condition. Natriuretic peptides, reflecting myocardial wall stress, are frequently elevated, although they may be normal at presentation, limiting their diagnostic value; nevertheless, elevated levels have been linked to worse prognosis [74].

Several emerging biomarkers have been investigated in myocarditis and infl-CMP, although their use remains largely confined to research or specialized centers. These include:

- MicroRNAs (miR-Chr8:96, miR-155, miR-206), which have demonstrated high specificity for myocardial inflammation. Serum levels of these miRNAs have been associated with the diagnosis of infl-CMP [75].
- Soluble ST2 (sST2), with elevated levels associated with more severe HF, particularly in younger male patients [76].
- Myocardial Galectin-3, correlating with inflammatory cell infiltration and inversely with fibrosis [77].
- Cardiac autoantibodies against structural or sarcolemmal proteins have been detected in several patients with persistent myocardial inflammation [11].
- Calprotectin (S100A8/A9) has recently emerged as an important mediator of myocardial inflammation through TLR-4 and RAGE signaling pathways. Recent studies have highlighted its role both as a mediator of cardiovascular disease and as a potential biomarker and therapeutic target [78,79]. In experimental models, inhibition of calprotectin has demonstrated beneficial effects. However, its clinical application as a biomarker and therapeutic target remains under investigation [80].

**Figure 1:**
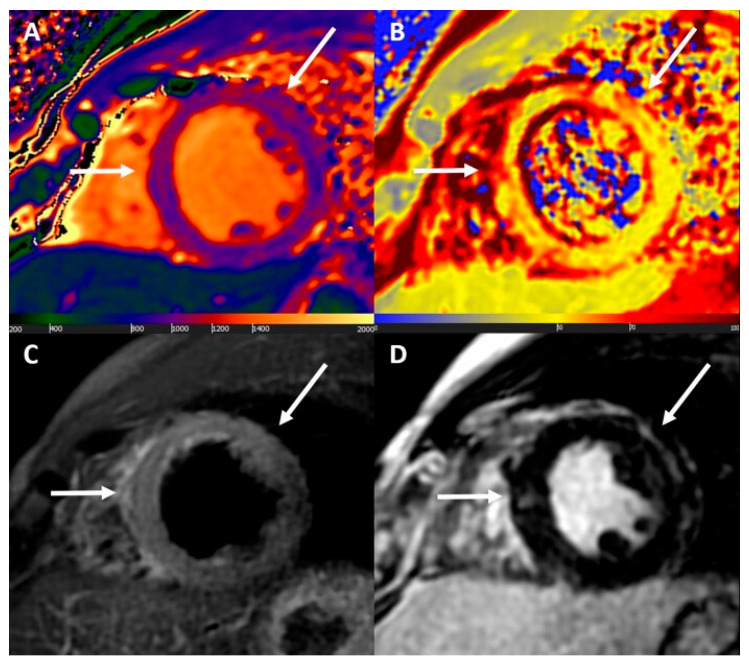
Cardiac magnetic resonance findings. Acute non-ischaemic myocardial injury at mid to apical septal, anterior, and anterolateral walls. (**A**) Increased native T1 values (up to 1260 ms); (**B**) increased T2 values (up to 71 ms); (**C**) increased signal intensity at T2-weighted images; and (**D**) nonischaemic late gadolinium enhancement. All abnormal findings have matched subepicardial distribution (white arrows). Reproduced with permission from ref. [81].

**Cardiovascular magnetic resonance.** The introduction of the modified Lake Louise criteria in 2018 significantly improved the sensitivity of myocarditis diagnosis using CMRI [44]. These criteria are based on at least one T2 (T2-weighted imaging or T2 mapping) and one T1 criterion (T1-mapping, extracellular volume, or LGE). The diagnosis is definitive if both criteria are present, while it is uncertain if only one criterion is present. Supporting criteria include the presence of pericardial abnormalities or LV systolic dysfunction [2]. Figure 1 illustrates the MRI findings in a patient diagnosed with myopericarditis recurrence after the third dose of BNT162b2 vaccine against SARS-CoV-2.

**Endomyocardial biopsy.** As regards EMB in addition to the Dallas histological criteria based on hematoxylin-eosin staining [82], immunohistochemical staining is used to identify CD3^+^ T cells and CD68^+^ macrophages, which are necessary to identify specific histological subtypes of myocarditis and differentiate them from phenocopies [47,83]. The Marburg criteria defined by ESC require the presence of ≥14 leukocytes/mm^2^, including ≥7 CD3^+^ T cells/mm^2^ [11]. Additionally, molecular analyses of EMB and blood (including the search for viral DNA and RNA in peripheral leukocytes and plasma) using polymerase chain reaction (PCR) are required when clinically useful to define the etiopathogenesis of myocarditis and guide therapy (antiviral or immunosuppressive) [11,47,84]. In cases of acute systemic and cardiac viral infection, immunosuppressive therapy should be avoided [2]. Routine viral testing in myocardium is not indicated, although it was suggested in the 2013 ESC consensus for differentiating virus-positive (infectious) from virus-negative myocarditis [44].

Based on individual risk, a definitive diagnosis is made through CMR or EMB. The current diagnostic algorithm first requires the exclusion of obstructive coronary artery disease, followed by the search for high-risk features that, if present, recommend the use of EMB (Class I, Level C). In cases of low risk, and prior to discharge in high-risk cases, CMR is indicated (Class I, Level B) [2]. Since oedema tends to resolve 4 weeks after the acute episode, CMR should be performed within 2 weeks of symptom onset, although accuracy may be lower in the first few days [44].

Current recommendations supporting the use of EMB in high-risk myocarditis or hemodynamically unstable patients, as well as in patients with intermediate-risk myocarditis not responding to standard treatments, rely mainly on expert consensus and limited clinical studies (Class I, Level C) [2]. EMB enables the early identification of patients who may benefit from immunosuppressive therapy by characterizing the histological subtype and determining the presence or absence of viral genomes. Additionally, biopsy is indicated in suspected myocarditis due to ICIs, given the poor sensitivity of echocardiography and CMR. EMB plays a critical role in rapidly progressive forms of myocarditis—such as FM, GCM, and EM—by allowing for the timely initiation of immunosuppressive treatment and improving outcomes [49]. In such cases, early EMB has been shown to be independently associated with a lower risk of death, heart transplantation/left ventricular assist device (LVAD) at 1 year [49].

Evidence suggests that the use of immunosuppressive therapy is associated with better outcomes and persistent improvements in cardiac function, including LVEF and LV dimensions, in cases of virus-negative infl-CMP [85]. Long-term functional recovery has been associated with the histological normalization of inflammatory processes [86]. Similar benefits have been reported in patients with AM accompanied by HF, systemic autoimmune diseases, or recurrent episodes of chest pain with troponin elevation, independent of histological subtype or clinical presentation, provided that viral genomes have been excluded [84]. The combination of prednisone and azathioprine represents the most frequently employed immunosuppressive regimen across both observational and interventional studies [84].

EMB is performed under fluoroscopic or electroanatomical guidance using a bioptome introduced via a transvenous approach (typically through the right internal jugular or femoral vein) or, in the case of LV biopsy, via arterial access. The most common site for EMB is the right ventricular septum, though sometimes LV or biventricular EMB may be required. The site decision should be based on the clinical indication, the operator’s expertise, and findings of the pre-procedural imaging [87]. Biventricular EMB can increase diagnostic accuracy compared with selective LV or RV EMB [88]. Current indications for biopsy require at least 3 samples. Given the focal distribution of lesions, especially in CS, the use of CMR or electroanatomical mapping (EAM) to guide biopsy may increase sensitivity [89] (Class IIa) [2].

**Figure 2:**
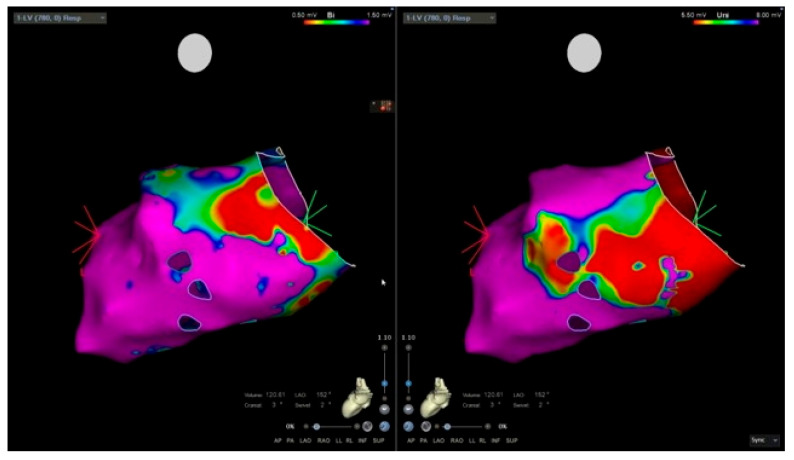
Electro-anatomic mapping and endomyocardial biopsy. Electro-anatomical voltage mapping of the left ventricle. The bipolar mapping is mainly normal (purple); low-voltage area (red) is noted in the inferolateral basal wall.

EAM, routinely used in our center to guide EMB, has demonstrated good sensitivity and specificity compared with CMR for the identification of pathological myocardial regions [90]. Its diagnostic utility in AM, however, may be limited, as low-voltage areas correlate well with LGE but not with myocardial oedema [91]. In some cases, a mismatch between low-voltage regions and LGE distribution on CMR may also be present. Nevertheless, the combination of unipolar and bipolar EAM with CMR improves the diagnostic yield of EMB [90], particularly in patients with CS [89]. The analysis of unipolar electroanatomical voltage mapping (EVM) has proven to correlate with epicardial pathological involvement, commonly observed in ARVC and myocarditis [92]. Figure 2 illustrates an electroanatomically guided endomyocardial biopsy.

Furthermore, EAM is useful in the differential diagnosis, especially for distinguishing ARVC from CS [93].

The complication rate associated with electroanatomically guided biopsy is <5% [89,94]. However, the occurrence of complications largely depends on the experience of the center, and the time required for sample analysis may represent a limitation in rapidly progressive conditions such as FM. Furthermore, although initiation of immunosuppressive therapy requires prior exclusion of viral genomes through PCR analysis, EMB findings are not always conclusive, and it can sometimes be difficult to distinguish whether viruses play a causative role in myocarditis or are merely bystanders.

## 6. Treatment

According to the 2013 ESC consensus paper, hospital admission was recommended for all patients with suspected myocarditis, including those who were asymptomatic or only mildly symptomatic [11]. The 2025 ESC guidelines update previous recommendations by advising hospital admission for patients with moderate-to-high-risk myocarditis (Class I, Level C) and suggesting it be considered for those at low risk (Class IIa) [2].

The goal of treatment is to alleviate symptoms and prevent complications, particularly recurrences and mortality in complicated cases. The targets of medical therapy include: infectious agents, inflammation, immune-mediated processes, HF and ventricular dysfunction, and arrhythmias. The 2025 ESC guidelines emphasize the close correlation between myocardial and pericardial diseases, introducing the umbrella term “inflammatory myopericardial syndrome” (IMPS). However, the treatment guidelines tend to focus on isolated cases of myocarditis and pericarditis. For combined conditions, the more relevant one (myopericarditis or perimyocarditis) should guide therapeutic choices and subsequent follow-up [2].

**Physical activity restriction.** A key component of non-pharmacological treatment is restriction of physical activity until symptom resolution and normalization of inflammatory markers. Depending on the clinical presentation, at least 1 month is required for clinical remission, but this may be prolonged [2]. According to the ESC 2020 guidelines on sports cardiology and exercise in patients with cardiovascular disease, resumption of sports activity should be considered in asymptomatic patients, with no evidence of ongoing inflammation, absence of arrhythmias, and normal LV systolic function, after 3–6 months from the event [95]. The current European guidelines [2], supported by the 2020 ACC consensus paper, suggest personalizing treatment until clinical remission is achieved, based on a multiparametric evaluation [96]. Clinical remission is defined as complete resolution of symptoms, normalization of troponin and CRP, ECG, echocardiography, and inflammation on CMRI.

**Pharmacological therapy**. Evidence on pharmacological treatment of myocarditis, particularly non-etiological therapy, remains limited and is primarily based on expert opinion or small clinical trials. Medical therapy is based on clinical presentation, severity, and etiology, and includes supportive care, non-etiological treatment (e.g., HF therapy in patients with systolic dysfunction) [97,98], antiarrhythmic therapy [9], and specific treatment. HF therapy should be continued for at least 6 months until complete recovery of LV function (Class IIa, Level C) [2]. Beta-blocker therapy should be considered in patients with AM and should be continued for at least 6 months (Class IIa, Level C) [2].

In patients with AM presenting with hemodynamic instability, prompt stabilization of the circulatory status is of paramount importance. In such cases, inotropes or temporary mechanical circulatory support (t-MCS) devices, including intra-aortic balloon pump, Impella, or VA-ECMO, may be required to maintain adequate perfusion.

Current guidelines indicate the use of non-steroidal anti-inflammatory drugs (NSAIDs) (Class IIa, Level C) in patients with associated pericarditis symptoms and colchicine in patients with myopericarditis to reduce recurrences [2,99]. NSAIDs may be used empirically in patients with uncomplicated myocarditis for chest pain control [100]. Immunosuppression is recommended in patients with specific subtypes of AM, such as EM, GCM, associated with systemic autoimmune disorders and ICI-induced myocarditis. Combined immunosuppressive therapy is recommended in patients with a diagnosed GCM (Class I, Level C) [2]. To date, no specific pharmacological therapies are recommended for the treatment of acute lymphocytic myocarditis beyond supportive management with inotropes and t-MCS.

Large registries frequently report the use of corticosteroids in cases of AM complicated by acute HF or cardiogenic shock, despite the current lack of evidence supporting this therapeutic strategy. The studies currently available mostly concern the use of immunosuppressive therapy at a later stage, such as in infl-CMP or AM with left ventricular dilation. Given the autoimmune-mediated mechanism of myocardial injury, it is conceivable that early therapeutic intervention might positively influence patient outcomes.

An ongoing trial is evaluating the efficacy and safety of high-dose intravenous corticosteroids in patients with acute or fulminant myocarditis based on clinical suspicion (ClinicalTrials.gov Identifier: NCT05150704).

Growing evidence has demonstrated significant activation of the NLRP3 inflammasome in the myocardium of patients with AM, suggesting a potential therapeutic target during the acute phase. NLRP3 activation triggers a rapid inflammatory cascade via IL-1β and IL-18 release. Consequently, anti-IL-1 agents (such as anakinra, rilonacept, and canakinumab) and colchicine have been proposed as promising therapeutic options, although clinical data remain limited [101].

Preliminary human studies with anakinra, an IL-1 receptor antagonist, have shown improvement in selected refractory cases of AM. However, the ARAMIS trial failed to demonstrate a significant clinical benefit in patients with suspected myocarditis without a specific aetiology, despite confirming the drug’s safety profile (ClinicalTrials.gov Identifier: NCT03018834) [102].

The role of intravenous immunoglobulins (IVIG) also remains under investigation. While retrospective studies and case series in adults suggest a potential improvement in transplant-free survival, robust randomized data are still lacking. IVIG therapy, however, is commonly employed in pediatric myocarditis [103].

## 7. Follow-Up

Patients with low-risk myocarditis may be discharged once myocardial necrosis enzymes tend to normalize. Healing times vary from a few days to a few months. Recurrences occur in 10% of cases, with a recurrence rate of 5% within the first year [5,11,104]. Follow-up is recommended for all patients with myocarditis and includes clinical reevaluation, biomarker assessment, ECG, exercise testing, Holter-ECG, echocardiography, and CMR at least within 6 months of hospitalization (Class I, Level C) [2,27]. Cardiopulmonary exercise testing (CPET) is used as the gold standard in our center for the follow-up of patients with a history of AM. In addition to assessing arrhythmic burden, CPET provides a comprehensive evaluation of functional capacity and enables a dynamic assessment of the cardiopulmonary system under physiologic stress, which is particularly valuable when evaluating fitness for safe return to exercise or competitive sports. Several CPET-derived parameters offer particularly informative insights:

- Peak oxygen consumption (VO_2_peak) is the primary indicator of cardiopulmonary fitness. Persistently reduced values may reflect impaired stroke volume, chronotropic incompetence, or physical deconditioning.
- Ventilatory efficiency (VE/VCO_2_ slope) reflects the effectiveness of ventilation relative to carbon dioxide production. VO_2_peak and VE/VCO_2_ slope are currently the most studied CPET variables in clinical settings, and both have demonstrated substantial, independent prognostic value in several cardiovascular diseases.
- Oxygen pulse (VO_2_/HR): The shape and magnitude of the oxygen pulse curve provide an indirect insight into stroke volume augmentation during exercise.
- Heart rate response and recovery: chronotropic incompetence and delayed heart rate recovery are markers of autonomic dysfunction and may persist after an episode of myocarditis.

CPET represents a safe and informative tool for evaluating functional recovery in patients with a history of AM. By enabling an objective assessment of integrated cardiopulmonary performance under physiologic stress, CPET facilitates the identification of subclinical exercise limitations and exertion-induced arrhythmias, thereby playing a central role in risk stratification and in determining appropriateness for resumption of physical activity. Despite its potential clinical value, no high-quality studies to date have specifically investigated the role of CPET in the follow-up of myocarditis. Most available data focus on the assessment of cardiac involvement following SARS-CoV-2 infection [105,106]. Nevertheless, considering the comprehensive physiological data provided by CPET, its inclusion in the follow-up assessment of myocarditis appears both reasonable and potentially beneficial.

Prognosis is determined by LV function at presentation and at 6 months, regardless of the clinical presentation [5,6,50]. Long-term follow-up is recommended for patients with FM (Class I, Level C) [2,3,107], while for uncomplicated myocarditis, follow-up at 6, 12, and 24 months is adequate [2].

**ICD implantation.** The arrhythmic risk of patients with myocarditis over time remains poorly known. It is generally accepted to wait 3–6 months after the acute episode to assess the indication for ICD implantation. In patients with sustained VA during the acute phase and a high risk of VA [108], the use of a wearable cardioverter-defibrillator as a bridge to recovery should be considered (Class IIa, Level C) [9]. ICD implantation for secondary prevention in patients with sustained VA (ventricular tachycardia (VT)/ventricular fibrillation (VF)) in the acute phase may be considered (Class IIb, Level C) [2]. In a multicenter registry of patients with AM, recurrences of VA occurred in approximately 3–9% of cases over a period of 19–90 months [3]. EAM may be a useful tool to identify patients at greater arrhythmic risk since the extension of the areas of low-potentials at the bipolar endocardial mapping has been associated with appropriate ICD interventions [109]. Further studies could investigate the possible prognostic role of the EVM in patients with myocarditis and moderate or preserved ventricular function, for which ICD in primary prevention is currently not indicated.

ICD implantation is indicated (Class I, Level C) in patients with inactive myocarditis and hemodynamically intolerated sustained VT, while it should be considered they are hemodynamically tolerated (Class IIa, Level C) [2].

After 3–6 months of follow-up, ICD implantation for primary prevention is decided based on the patient’s individual risk, considering the presence of non-sustained ventricular arrhythmias, extended LGE on CMR, unexplained syncope, positive programmed ventricular stimulation (PVC), and LVEF < 50%, while also taking into account genetic predisposition [2].

## 8. Conclusions

Knowledge about myocarditis has evolved significantly in recent years. Multiple etiologies have been identified, including viral and toxic causes, such as chemotherapy, as well as systemic disorders, with tailored therapeutic approaches now being proposed.

A major diagnostic paradigm change is the capability of reaching a clinical diagnosis of certainty by means of non-invasive multimodality imaging (CMR for myocarditis), while EMB is reserved for high- and intermediate-risk cases, with the aim of guiding specific treatment strategies.

Most cases of myocarditis follow an uncomplicated course, and treatment is empirical, aiming at the control of symptoms. Myocarditis-related complications are relatively rare; as a result, clinical trials designed to assess the benefit of specific therapies may be underpowered due to the limited size of the study populations. Immunosuppressive therapy is recommended in specific histological subtypes, such as EM and GCM, as well as in cases of myocarditis associated with systemic autoimmune diseases. However, strong evidence supporting the use of specific therapies in AM complicated by acute HF or cardiogenic shock is still lacking and is currently being investigated.

The role of genetic predisposition in recurrent myocarditis remains to be clarified, and an increase in overlap cases with cardiomyopathies—particularly dilated cardiomyopathy (DCM) and arrhythmogenic right ventricular cardiomyopathy (ARVC)—can be expected.

Despite advances in diagnostic and therapeutic strategies, substantial evidence gaps persist in the field of myocarditis. The majority of current recommendations outlined in recent European guidelines remain grounded in small-scale studies or expert consensus. There is a pressing need for high-quality evidence to inform both the acute management of myocarditis—including the role of EMB and immunosuppressive therapies across the full clinical spectrum—as well as long-term risk stratification, particularly with regard to the identification of patients at increased arrhythmic risk and the selection criteria for ICD implantation.

## Figures and Tables

**Table 1:** Terminology and stages.

Terminology	Definition
Acute myocarditis	Duration of symptoms ≤ 4 weeks Fulminant myocarditis is characterized by acute onset and hemodynamic instability, requiring inotropic therapy or mechanical circulatory support.
Subacute/ongoing myocarditis	Duration of symptoms > 4 weeks to ≤3 months
Chronic myocarditis	Duration of symptoms > 3 months
Recurrent myocarditis	New symptoms or disease activity after clinical remission
Inflammatory cardiomyopathy	Chronic myocarditis associated with cardiac dysfunction and ventricular remodeling with clinical phenotype of hypokinetic, either dilated or non-dilated cardiomyopathy with/without arrhythmogenic substrate
Complicated myocarditis	Acute myocarditis and ≥1 of the following: LVEF < 50% on echocardiogram Sustained ventricular arrhythmias Advanced heart block HF Cardiogenic shock
Remission with residuals	Regression or absence of symptoms, with persistence of abnormalities on ECG, biomarkers, and/or imaging, including functional or structural alterations detected by echocardiography or CMR.
Remission without residuals	Regression or absence of symptoms, normalization of ECG, biomarkers, and imaging findings, including echocardiography and CMR.

CMR: cardiovascular magnetic resonance; ECG: electrocardiogram; HF: heart failure; LVEF: left ventricular ejection fraction.

**Table 2:** Clinical risk stratification.

**High Risk**	**Intermediate Risk**	**Low Risk**
Acute HF/cardiogenic shock Dyspnea NYHA III-IV refractory to medical therapy Cardiac arrest/syncope Ventricular fibrillation/sustained ventricular tachycardia High-level AV block	New/progressive dyspnea Non-sustained ventricular arrhythmias Persistent release or relapsing troponin	Stable symptoms or oligosymptomatic
**Imaging Criteria**	**Imaging Criteria**	**Imaging Criteria**
Newly reduced left ventricular ejection fraction (<40%)	Newly mildly reduced left ventricular ejection fraction (41–49%) and/or WMA	Preserved left ventricular ejection fraction (≥50%) without LGE or limited LGE (<2 segments) on CMR
Extensive LGE on CMR	Preserved LVEF (≥50%) and LGE ≥ 2 segments on CMR	

AV: atrioventricular; CMR: cardiovascular magnetic resonance; HF: heart failure; LGE: late gadolinium enhancement; WMA: wall motion abnormalities.

**Table 3:** Red flags for the clinical diagnosis of myocarditis.

Recent or concomitant flu-like syndrome or gastroenteritis Infarct-like chest pain Palpitations Symptoms of heart failure ECG changes Ventricular arrhythmias (isolated or complex) Syncope Hemodynamic instability Elevated markers of myocardial injury (hs-Tn, CK-MB) Elevated marker of heart failure (NT pro-BNP) Abnormal wall motion, increased wall thickness, and/or impaired systolic function on imaging CMR imaging evidence of myocardial oedema and/or LGE

CK-MB: creatinine kinase muscle-brain type; CMR: cardiovascular magnetic resonance; ECG: electrocardiogram; hs-Tn: high sensitivity troponin; LGE: late gadolinium enhancement; NT-proBNP: N-terminal prohormone of brain natriuretic peptide.

**Table 4:** Recommendations for genetic testing.

Recommendation
Obtaining a family history, including pedigrees, is recommended in cases of recurrent inflammatory myopericardial syndrome to help identify the underlying etiology, determine the pattern of inheritance, and recognize relatives at risk (Class I, Level C) Genetic testing should be considered in patients with definite myocarditis in the following situations (Class IIa, Level B): A family history of myocarditis or inherited/suspected cardiomyopathy Severe ventricular arrhythmias Significant left or right ventricular LGE (e.g., ring-like pattern or septal LGE) or persistent systolic dysfunction (reduced LVEF) Recurrent myocarditis or persistently elevated troponin

LGE: late gadolinium enhancement; LVEF: left ventricular ejection fraction.

**Table 5:** ESC 2013 diagnostic criteria for clinically suspected myocarditis.

Clinical Presentation
Acute chest pain, either pericarditic or pseudo-ischemic New-onset (days up to 3 months) or worsening dyspnea at rest or on exertion, and/or fatigue, with or without signs of left and/or right heart failure Subacute or chronic symptoms (>3 months), or worsening dyspnea and/or fatigue, with or without left and/or right heart failure signs Palpitations, unexplained arrhythmia symptoms, syncope, or aborted sudden cardiac death Unexplained cardiogenic shock
**Diagnostic Criteria**
I. **ECG, Holter, and stress test abnormalities** Newly abnormal 12 lead ECG, Holter and/or stress testing may include any of the following: first to third degree atrioventricular block, or bundle branch block, intraventricular conduction delay (widened QRS complex), ST-segment or T wave changes (ST elevation or non ST elevation, T wave inversion), reduced R wave amplitude, abnormal Q waves, low voltage, sinus arrest, ventricular tachycardia or fibrillation and asystole, frequent premature beats, supraventricular tachycardia, atrial fibrillation II. **Myocardiocytolysis markers** Elevated TnT/TnI III. **Functional and structural abnormalities on cardiac imaging (echocardiography/angiography/CMR)** Newly detected, otherwise unexplained left and/or right ventricular structural or functional abnormalities, including incidental findings in asymptomatic individuals: regional wall motion abnormalities, global systolic or diastolic dysfunction, with or without ventricular dilatation, increased wall thickness, pericardial effusion, or endocavitary thrombi IV. **Tissue characterization by means of CMR** Myocardial oedema and/or LGE in a classical myocarditic pattern

Clinically suspected myocarditis if ≥1 clinical presentation and ≥1 diagnostic criterion from different categories. If the patient is asymptomatic, ≥2 diagnostic criteria should be met. CMR: cardiovascular magnetic resonance; LGE: late gadolinium enhancement; Tn: troponin.

**Table 6:** ESC 2025 diagnostic criteria for myocarditis.

Definite	Possible	Unlikely/Rejected
Clinical presentation and CMR or EMB-proven	Clinical presentation with at least 1 additional criterion CMR- or EMB-uncertain or not available	Only clinical presentation without additional criteria

CMR: cardiovascular magnetic resonance; EMB: endomyocardial biopsy.

**Table 7:** Additional criteria beyond clinical presentations.

Clinical	Non-Specific Findings
ECG	ST-T changes
Biomarkers	Troponin elevation
Imaging	Abnormal strain, wall motion, reduced EF Myocardial oedema and/or LGE (CMR findings)

CMR: cardiovascular magnetic resonance; EF: ejection fraction; LGE: late gadolinium enhancement.

## Abbreviations

The following abbreviations are used in this manuscript:

ACC	American College of Cardiology
AM	Acute myocarditis
ARVC	Arrhythmogenic right ventricular cardiomyopathy
AVB	Atrioventricular block
B19V	Parvovirus B19
CK-MB	Creatinine-kinase muscle-brain type
CMR/CMRI	Cardiovascular magnetic resonance/cardiovascular magnetic resonance imaging
COVID-19	Coronavirus disease 19
CPET	Cardiopulmonary exercise testing
CRP	C-reactive protein
CTLA-4	Cytotoxic T-lymphocyte antigen 4
CS	Cardiac sarcoidosis
DCM	Dilated cardiomyopathy
DNA	Deoxyribonucleic acid
DSG	Desmoglein
DSP	Desmoplakin
EAM	Electroanatomical mapping
EBV	Epstein–Barr virus
ECG	Electrocardiogram
EMB	Endomyocardial biopsy
EM	Eosinophilic myocarditis
ESC	European Society of Cardiology
ESR	Erythrocyte sedimentation rate
EVM	Electroanatomical voltage mapping
FM	Fulminant myocarditis
GCM	Giant cell myocarditis
HF	Heart failure
HHV-6	Human herpesvirus 6
hs-Tn	High-sensitivity troponin
ICD	Implantable cardioverter defibrillator
ICI	Immune checkpoint inhibitor
IMPS	Inflammatory myopericardial syndrome
infl-CMP	Inflammatory cardiomyopathy
IVIG	Intravenous immunoglobulins
LGE	Late gadolinium enhancement
LV	Left ventricle/left ventricular
LVAD	Left ventricular assist device
LVEF	Left ventricular ejection fraction
NGS	Next-generation sequencing
NSAID	Non-steroidal anti-inflammatory drug
PCR	Polymerase chain reaction
PD-1	Programmed cell death protein 1
PDL-1	Programmed cell death ligand 1
PVC	Programmed ventricular stimulation
RAGE	Receptor for advanced glycation end-products
RNA	Ribonucleic acid
RV	Right ventricle
SCD	Sudden cardiac death
ST	ST segment
TLR	Toll-like receptor
t-MCS	Temporary mechanical circulatory support
TnI/TnT	Troponin I/troponin T
TTN	Titin
VA	Ventricular arrhythmias
VA-ECMO	Venoarterial extracorporeal membrane oxygenation
VO_2_peak	Peak oxygen consumption
VE/VCO_2_ slope	Minute ventilation/carbon dioxide production slope
VO_2_/HR	Oxygen pulse (oxygen consumption per heart rate)
VF	Ventricular fibrillation
VT	Ventricular tachycardia
WMA	Wall motion abnormalities
